# A Comparison between the Decimated Padé Approximant and Decimated Signal Diagonalization Methods for Leak Detection in Pipelines Equipped with Pressure Sensors

**DOI:** 10.3390/s18061810

**Published:** 2018-06-04

**Authors:** Aimé Lay-Ekuakille, Laura Fabbiano, Gaetano Vacca, Joël Kidiamboko Kitoko, Patrice Bibala Kulapa, Vito Telesca

**Affiliations:** 1 Department of Innovation Engineering, University of Salento, 73100 Lecce, Italy; 2 Department of Mechanics Mathematics & Management, Polytechnic of Bari, 70100 Bari, Italy; laura.fabbiano@poliba.it (L.F.); gaetano.vacca@poliba.it (G.V.); 3 Faculty of Engineering, Bel Campus University of Technology, 01 Kinshasa, DR Congo; joekid012@gmail.com; 4 Department of Electronics, ISTA University, 01 Kinshasa, DR Congo; patricebibala@gmail.com; 5 School of Engineering, University of Basilicata, 85100 Potenza, Italy; vitotelesca64@gmail.com

**Keywords:** detecting leakage in pipelines, revealing cracks in waterworks, pressure sensors, nondestructive tests, structural health monitoring

## Abstract

Pipelines conveying fluids are considered strategic infrastructures to be protected and maintained. They generally serve for transportation of important fluids such as drinkable water, waste water, oil, gas, chemicals, etc. Monitoring and continuous testing, especially on-line, are necessary to assess the condition of pipelines. The paper presents findings related to a comparison between two spectral response algorithms based on the decimated signal diagonalization (DSD) and decimated Padé approximant (DPA) techniques that allow to one to process signals delivered by pressure sensors mounted on an experimental pipeline.

## 1. Introduction

Nondestructive methods are useful for structural health monitoring (SHM) which is an important field of research and development. Among important structures and infrastructures, we include pipelines conveying fluids in aqueous and gaseous states, respectively. In particular, pipelines for water, especially waterworks, must be under control when they are located outside the city, in this case, they are called primary pipelines. For drinkable water distribution within a city, the issue is basically strategic, and the quality of pipe along with its buried location are factors to be taken into account. The process of implementing a damage identification strategy for aerospace, civil and mechanical engineering infrastructure is referred to as SHM. This process involves the observation of a structure or mechanical system over time using periodically spaced measurements, the extraction of damage-sensitive features from these measurements, and the statistical analysis of these features to determine the current state of system health. For long-term SHM, the output of this process is periodically updated with information regarding the ability of the structure to continue to perform its intended function in light of the inevitable aging and damage accumulation resulting from the operational environments. The efficiency of most waterworks is not generally high because of losses, that is leakage. 

**Figure 1:**
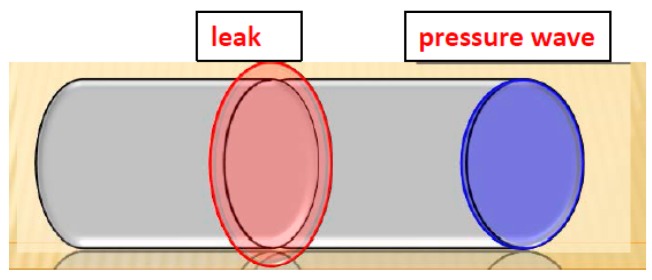
Displaying leakage and pressure wave.

The water resources community has perhaps focused more on the natural environment in the past but as a matter of fact, the protection of water quality against intrusion in pipes or defense of the environment from the release of a conveyed pollutant is as meaningful as the defense of well-fields and aquifers from pollutant release [1]. A leak in a pipeline, as illustrated in Figure 1, causes partial reflections of wave fronts that become small pressure discontinuities in the original pressure trace and increase the damping of the overall pressure signal. Such partial reflections act to divert energy away from the main waveform and increase the decay rate of the transient signal. The behavior of this pressure trace is, therefore, indicative of leaks within the system and can be used as a means of leak detection. There are different reasons that may cause leaks: (i) quality of the materials used for the pipeline: e.g., polyurethane/polyethylene, metal, stoneware, synthetic; (ii) junctions, and valves. All envisaged methods have the main scope to detect these reasons. They are also considered as on-line nondestructive methods since they preserve the pipeline.

**Figure 2:**
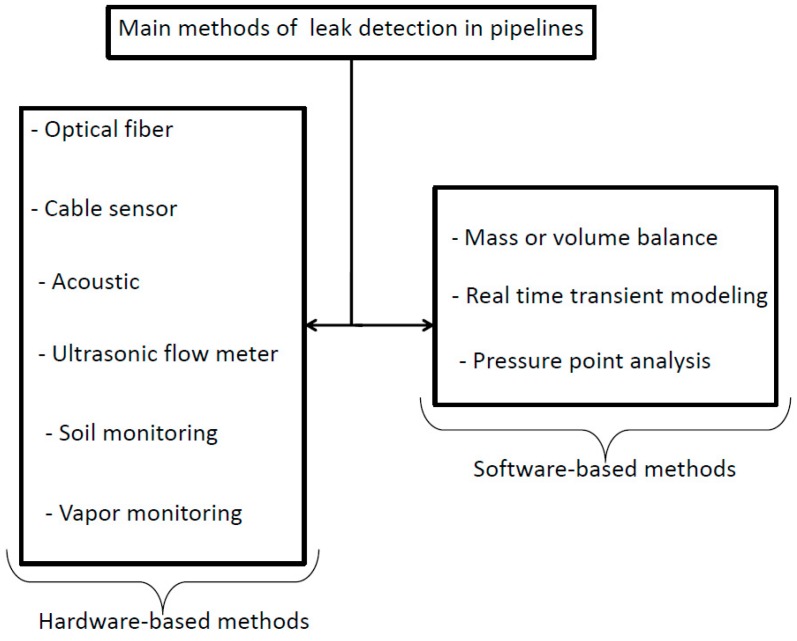
General classification of leak detection methods.

According to a broad classification, there are various methods for retrieving leakage in pipelines, as illustrated in Figure 2. Hardware-based methods are related to dedicated instrumentation and components to be used for monitoring water flow within the pipeline. They can be fixed on the pipe or they are portable. On the other hand, software-based methods focus on data and/or signal processing. However, some of software-based methods use inverse methods to determine parameters in transient models by comparison with observed data (inverse transient analysis), transient damping-free-vibrational analysis [2], and also methods that utilize the time of arrival and magnitude of leak-reflected signals to determine leak location [3]. All these published fluid transient leak detection methods share a common theme in that a small amplitude disturbance—a fluid transient—is initiated in a pipe and the subsequent pressure response is measured and analyzed to derive system information. This type of analysis is more commonly known as system response extraction [4] and forms the basis of well established methodologies used to extract dynamic responses of complex mechanical and electrical systems.

## 2. Pressure Sensor Technology for Liquid Control 

Leak detection in pipelines is an example of piezoelectric application (pressure sensing generally uses the piezoelectric effect) for SHM, and whatever signal processing technique we can adopt, as the impact of the pressure sensing device efficiency is also definitely connected to the performance of the piezoelectric sensor. There are pressure sensors based on the piezoelectric effect, that can work with high sensitivity, due to the quality of materials used for these purposes. They are realized in reinforced fiber [5], that is, piezo fiber composites. Moreover, further SHM monitoring can be obtained using conventional 5H PZT (lead zirconate titanate) piezoceramics, piezoelectric diaphragms, and macro fiber composite (MFC) devices, and that react with a creaking in case of crack and/or defect within the structure to be monitored [6]. These devices are suitable, as said before, for cracks to be repaired, and that not require immediate intervention as in the case of pipelines; we would mean, the sensors used in this paper are piezoelectric but the goal is a continuous monitoring of the integrity of the pipe but we exploit them for putting under control the normal flow of the pipeline. New piezoelectric pressure devices, based on dedicated materials, known as “smart patches” [7], can be used for SHM and structural repair. Their main and exceptional advantages consist in repairing, self-rehabilitating, and self-vibrating damping. These features are important for monitoring and repairing cracks provoking leaks on pipelines. These features also depend upon the type of materials. 

However, an evolution of pressure sensors has been noticed so that they can be used for liquids and gases, thanks to specific technologies. As aforementioned, PZT, for example, encompasses poled polycrystalline ceramics [8]. The lead zirconate titanate (PbZr_1−x_ Ti_x_O_3_, PZT) system employs thin film delivering piezoelectric and dielectric properties. Their structures are in perovskite (ABO_3_) in cubic, rhombohedral, and orthorhombic shapes. They are subject to composition and temperature.

Unsophisticated but excellent technologies for pressure sensors are implemented using the following features: (i) bonded foil strain gage, used for heavy fluids detection. It is generally based on Wheatstone bridges; (ii) the bonded foil strain gauge can be improved using silicon piezoresistive embedded in a silicon diaphragm. This constructive configuration allows an enhancement of output signal in presence of lower pressure ranges, then, high sensitivity for pressure inputs. A detailed and comparative framework of pressure sensors is illustrated in [9] where a special focus is devoted to SIC-based and SOI-based sensors. They are very useful for SHM. 

## 3. DPA and DSD Techniques

As we have seen in the aforementioned section, there are high performing sensors and transducers that can be easily used for the scope of this paper, in general in SHM; sensors, per se, can only deliver the pressure value, a negative one in the case of a leak, but not the position of the leak in a pipe. That is done thanks to dedicated algorithms with or without necessary hardware. Advanced sensors performances reduce noise. However, it is necessary to clarify the fact that recovering spectra in frequency domain is a key issue in leak detection based on signal processing. Moreover, two general constrains weigh on the performances of spectrum retrieval: amount of data to be processed, then risk of information loss, and the time requested to compute the processing. That is why advanced transforms used in nuclear magnetic resonance are interesting, and two of them are details in this section. Decimated Padé Approximant (DPA) [10] and Decimated Signal Diagonalization (DSD) [11] belong to software-based methods as reported in the previous section. To simplify the mathematical procedures, we start with DSD and after that it will be easy to understand DPA. The advanced transforms, namely DSD and DPA, are used here as spectroscopic methods to find interested spectra related to peaks, and corresponding leaks. DSD, introduced by [12], is another way, as alternative, for signal processing with high resolution delivering an equidistant grid with a narrow convolution. 

DSD has been created for working on part of the filter diagonalization method (FDM) [13] making processing easier, and allowing one to build small matrices of a generalized eigenvalue problem using digitized points of the band-limited decimated signal. Given *K* as the length of the signal less than +∞, *n* a parameter indicating the current sample with a maximum of *N* − 1 as reported below. It is thought for signals with the following analytical description:

(1) $$c_{n}=\sum_{k=1}^{K}d_{k}e^{-j\omega_{k}n\tau }$$

That means, signals that can be expressed as summation of damped exponentials with complex amplitude *d_k_* and frequency *ω_k_*. It is important to mention that the previous formula is a nonlinear fitting, different from the linear fitting of discrete IFT (inverse Fourier transform). In IFT, frequencies are purely real and exponentials are not damped; these frequencies are determined by the problem parameters and they correspond to equidistant points $\omega_{k}=\frac{2\pi_{}}{\tau }\frac{k}{N}$, to which there is a resolution in fixed frequency Δf=1/Nτ. On the contrary, in nonlinear fitting, a significant advantage is displayed because of the increase of resolution. Let *c*(*n*), for n=0,…,N−1 be a signal with *N* acquired samples by means of sampling with step *τ*, having band 2π/τ. the first operation foreseen by DSD is to carry out FFT (fast Fourier transform) on signal *c*(*n*), by obtaining the spectrum:

(2) $$C_{k}=\sum_{n=0}^{N-1}c_{n}e^{j2\pi \frac{n}{N}k},\;\;k=0,\;\ldots ,\;N-1$$

which is a low resolution spectrum because the number of samples *N*, in general, is not sufficient to represent close frequency components. As we may know, applying such transformation means to assume that the interested signal is periodical with period equal to the length of the considered signal, since in this case the transformation is exact, but this condition does not occur, and then FFT filtering can only be approximated. This is a partial limitation (a few one) of DSD as a high resolution technique with respect to FDM, that, most likely, might allow best results for those frequencies close to edge of windows, in particular, for most narrow windows. The next step is to subdivide the FFT spectrum in appropriate number *M* of windows $\left[\omega_{k}{}_{\min},\;\omega_{k}{}_{\max}\right]$ so that the number $N_{D}=k_{\max}-k_{\min}+1$ of samples of any window must not be greater than a certain value. In fact, to limit *N_D_* (window of dimension *D*) towards low levels allows to reduce either the ill-posed problem or its dimension, and the computational cost to solve the problem.

It is important to notice the following remarks:

- it is not compulsory to work on windows of the same dimension.
- one can think to generate windows so that the adjacent ones can be partially overlapped and then proceed to the removal of portions constituted by overlapped windows in the effort to generate one more “fluid” spectrum; that is done during the step of spectral re-composition.
- in DSD, the windowing is fully separate from the spectral analysis, and single signals $\left\{c_{n}^{bld}\right\}$ can be analyzed in frequency with a method even different from the method of generalized eigenvalues. The superscript *bld* stands for band limited. However, in this paper we refer as to the method of generalized eigenvalues (MGEV). Conversely, MGEV is the only manner for FDM because there is a direct connection between windowing and MGEV. To simplify the dissertation, let us consider the simplest case in which the spectrum is divided in *M* = *N*/*N_D_* windows of the same dimension (*N_D_*), not overlapped with each other. After windowing, each windowed spectrum ${\left\{C_{k}^{bld}\right\}}_{k=0,\ldots ,N_{D}-1}$ is shifted for centering it with respect to the origin of frequencies ω=0; the value of the shifting is related to the position of the spectrum, and it is different for any of them. In absolute value, it is:
  (3) $$\omega_{0}=\frac{\pi (k_{\max}+k_{\min})}{N\tau }=\frac{2\pi k_{0}}{N\tau }$$

The *M* spectra have band-with equal to $\frac{2\pi }{\tau }\frac{N_{D}}{N}$, *M* times smaller than the inner spectrum, that is why they denoted as “band limited”. To go back to the time domain, we use the IFFT, anti-transforming spectra $\left\{C_{k}^{bld}\right\}$ for obtaining the *M* signals with limited band ${\left\{c_{n}^{bld}\right\}}_{k=0,\ldots ,N_{D}-1}$ having only *N_D_* samples.

The Padé approximant (PA), which is the parent of DPA, is the most important member of the whole family of nonlinear transformation denoted as sequence-to-sequence note. PA is the rational approximation mostly used in many fields. The main reason of reaffirming PA in physics is its mathematical equivalence with a certain number of principal methods of quantum mechanics; such as expansions of Born’s perturbations [14], finite rank expansions, separable exponential expansions, variational principles of Schwinger [15], Green’s functions [16], determinants of Fredholm [17], etc. The theorem of Weierstrass [18] states that, as a matter of principle, each real-valued continuous function on a given non-empty domain can be approximated by a polynomial with any necessary accuracy. However, in practice, many functions, with singularity in some areas of their definition domains, need to be approximated with diverse polynomials. One of the possibilities is to use a rational function; that is for example:

(4) $$f\left(z\right)\approx \frac{A_{L}\left(z\right)}{B_{K}\left(z\right)}$$

Here, *A_L_*(*z*) e *B_k_*(*z*) are generally polynomials of degree *L* and *K*, respectively:

(5) $$A_{L}\left(z\right)=\sum_{l=0}^{L}a_{l}z^{l}\;\;\;B_{K}(z)=\sum_{k=0}^{K}b_{k}z^{k}$$

in which the variable *z*, and coefficients {*a_l_*,*b_k_*} are complex values. The rational polynomial *A_L_*(*z*)/*B_K_*(*z*) forms a bidimensional table *L* × *K*, called “table of Padé”, that represents a set of functions of diverse degree *L*, and *K* respectively. An alternative method to solve nonlinear systems of the following type:

(6) $$c_{n}=\sum_{k=1}^{K}d_{k}z_{k}^{n}\;n=0,1,\ldots ,2K-1$$

is now proposed to apply DPA, where *d_k_* and *z* are respectively the amplitude and the variable under consideration. This method is the Padé approximating standard (PAS) but applied to a decimated signal with limited band. Assuming for now that points of the signal *c_n_* are known on an infinite number n=0,…,+∞ by interpreting coefficients of signal of shorter length as coefficients of the expansions in MacLaurin series using the variable *z*^−1^, then, we can define the function:

(7) $$g\left(z\right)=\sum_{n=0}^{\infty }c_{n}z^{-n}$$

applying a band-limited signal $c_{n}^{bld}$, and the Padé standard approximation is defined as a unique rational approximation with the following representation:

(8) $$\sum_{n=0}^{\infty }c_{n}^{bld}z^{-n}≅{\left[\frac{M}{K}\right]}_{f}\left(z\right)=\frac{\sum_{j=0}^{M}p_{j}z^{j}}{\sum_{j=0}^{K}q_{j}z^{j}}=\frac{P_{M}\left(z\right)}{Q_{K}\left(z\right)}$$

Notice that the series of exponents in (8) is the unilateral *z*-transform. We use the formulation of Padé that imposes the condition of normalization *Q_k_*(0) = 1. The remaining coefficients are chosen so that the series exponents of the approximant correspond to the series of exponents of the approximated function to degree *M* + *K* included. The Padé equations can be written as:

(9) $$\sum_{j=0}^{K}q_{j}c_{n-j}^{bld}=p_{n}\;\;\;\;n=0,\ldots ,M$$

(10) $$\sum_{j=0}^{K}q_{j}c_{n-j}^{bld}=0\;\;\;n=M+1,\ldots ,M+K;$$

in which if *n* < *j*, $c_{n}^{bld}$ = 0.

Coefficients of numerators and denominators can be computed by using the recursive algorithm of Wynn [19], or using directly the equations of Padé. Within the direct approach, they can be solved using single value decomposition (SVD [20] or lower-upper (LU) decomposition [21] with iterative refining to provide for denominator coefficients; thus, these latter have been replaced in Equation (9) to supply numerator. For *M* = *K* ± 1, and *M* = *K* we obtain para-diagonal approximants $P_{k\pm 1}(z)/Q_{k}(z)$, and the diagonal approximants $P_{k}(z)/Q_{k}(z)$, respectively.

Empirically speaking, it was noticed that approximants deliver the minimum error for a particular *M* + *K*, and they are just these approximants that are used in the DPA process. The polynomial of denominator $Q_{k}(z)$ is the characteristic formula of differences equations. Poles of approximant are given by $Q_{k}(z_{k})$, $z_{k}=\exp(-i\omega_{k}T_{d})$, *k* = 1, …, *K*. When we use polynomials of order greater than 60 (as empirical consideration), it would be appropriate for a top diagonalization of Hessenberg matrix like:

(11) $$Q=\left(\begin{array}{cccc} -\frac{q_{K-1}}{q_{K}} & -\frac{q_{K-2}}{q_{K}} & \begin{array}{cc} \cdots & -\frac{q_{1}}{q_{K}} \end{array} & -\frac{q_{0}}{q_{K}}\\ 1 & 0 & \begin{array}{cc} \cdots & 0 \end{array} & 0\\ \begin{array}{c} 0\\ \vdots \end{array} & \begin{array}{c} 1\\ \ddots \end{array} & \begin{array}{c} \begin{array}{cc} \ddots & 0 \end{array}\\ \begin{array}{cc} \ddots & \ddots \end{array} \end{array} & \begin{array}{c} 0\\ \vdots \end{array}\\ 0 & \cdots & \begin{array}{cc} 0 & 1 \end{array} & 0 \end{array}\right)$$

Complex frequencies can be computed from poles, using:

(12) $$\omega_{k}=\frac{i}{T_{d}}\log(z_{k})$$

in which $\ln\left(z_{k}\right)=\ln\left|z_{k}\right|+iArg\left(z_{k}\right)$. Assuming that roots of $Q_{k}\left(z_{k}\right)$ distinct, complex amplitudes can be found by using the so-called formula of Cauchy’s residues theorem [22], that yields to:

(13) $$d_{k}=\frac{P_{K}\left(z_{k}\right)}{z_{k}Q_{K}^{\prime }\left(z_{k}\right)}\;\mathrm{or}\;d_{k}=\frac{P_{K}\left(z_{k}\right)}{Q_{K}^{\prime }\left(z_{k}\right)}$$

with input the series $\sum_{n=0}^{\infty }z^{-n-1}$, and $\sum_{n=0}^{\infty }z^{-n}$, respectively.

The extraction of amplitudes, in this way, is more accurate and precise, and computationally efficient. Moreover, Cauchy’s residues theorem, for multiple poles, offers the possibility to work with overlapped spectral features. After explaining both methods in a mathematical viewpoint, it is important to understand the reasons of their use in leak detection with piezoelectric sensors in particular, and signal processing in general. Contrary to traditional spectral analysis such as FFT, based on nonlinear estimation of parameters, that processes sums and integrals, both DPA and DSD have the goal to solve an algebraic system by means of the search of eigenvalues and eigenvectors of the associated matrix, with which it is necessary to calculate parameters of amplitude and frequency of spectral components. DSD is the method, computationally speaking, easy and with major calculation speed, and with the best signal estimation; conversely, DPA exhibits a major resolution with a spectrum with more information but computationally “complex”. In fact, a major complexity corresponds to a major time of processing. Practically speaking, a complicated pipeline with different and accentuated heights (piezometric gaps) which leaks information can be easily processed with DPA whether we need details on the position and additional information; but it requires more time than that asked for processing with DSD. To be precise, and according to our experience, given the same length, the difference in terms of computation time in around 30–40% detrimental for DSD. Analogously, in case of pipeline located in a plane, with less curves, DSD would be better than DPA, because it would exhibit high speed in processing. This sentence can be intended in other words: DSD (decimated signal diagonalization) truncates part of the spectrum to accelerate the process.

## 4. Experimental Aspects and Results

**Figure 3:**
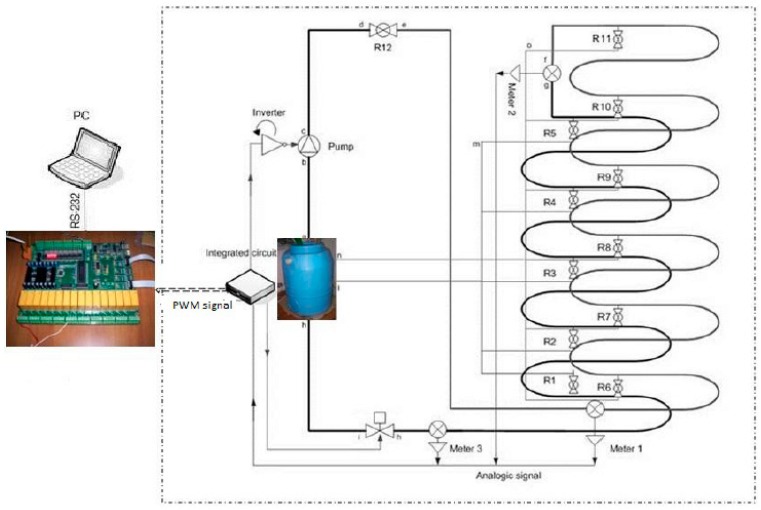

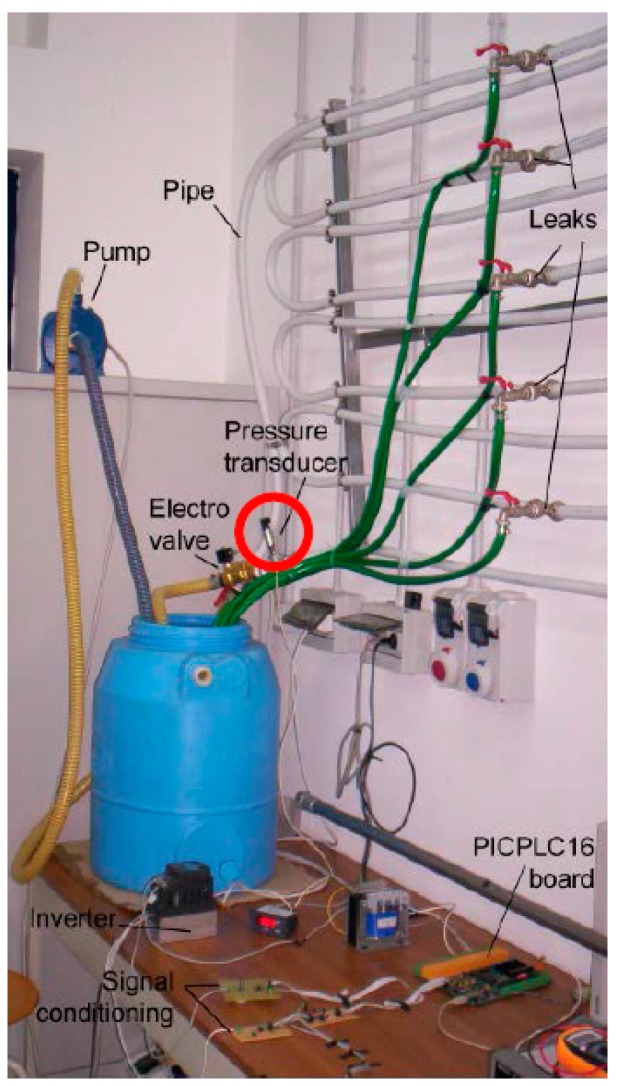
Experimental plant with three pressure sensors: (**top**) architecture and (**bottom**) real plant with circled pressure sensor.

The research activities as well as experimental measurements have been performing on real plant located in the laboratory of Measurements and Instrumentation of the Department of Innovation Engineering of the University of Salento. It is a 120 m (1 inch section) zigzag pipeline hung on the wall of the laboratory, simulating severe conditions of a pipeline. Three pressure sensors are located on the pipeline at specific distances. It is a metal−based pipe in copper with a double layer. The main components of the hydraulic plant are illustrated in Figure 3 and are the following: a tank with water, a pump electrically connected to an inverter for speed variations, an electrovalve, eleven water taps for simulating leakage, a dedicated electronics, a PICPLC16B board for controlling input/output signal and connected to a supervising computer. Many tests have been performed to establish the correct operating modes to acquire signals and to discriminate leaks from sudden variation of pressure [23]. 

**Figure 4:**
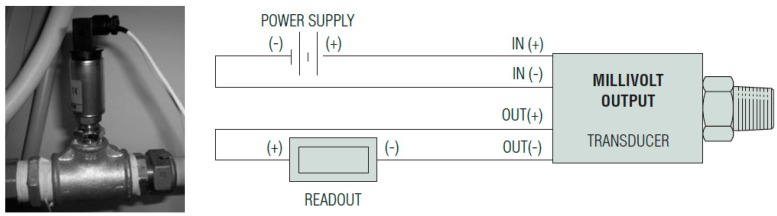
One of the pressure transducer/sensor (Gems 2200) located on the pipeline with its positioning (**left**) and technical features (**right**).

Figure 4 indicates the pressure sensor [24,25] here used as pressure device for detecting features of the water flow within the pipeline. It is a strain gauge CVD technology that allows an accuracy of 0.25% with an amplifier unit (ASIC). CVD stands for chemical vapor deposition, and details and drawings are illustrated in [26]. The proposed approach must be considered as nondestructive test of pipelines and could be extended to other infrastructures. The sensor detects the pressure variation, and monitors it in a continuous way. The voltage coming from the device represents the pressure of the flow within the pipeline. Values of voltage are acquired by a PICPLC16B board and are processed according to the two algorithms proposed in this paper. 

**Figure 5:**
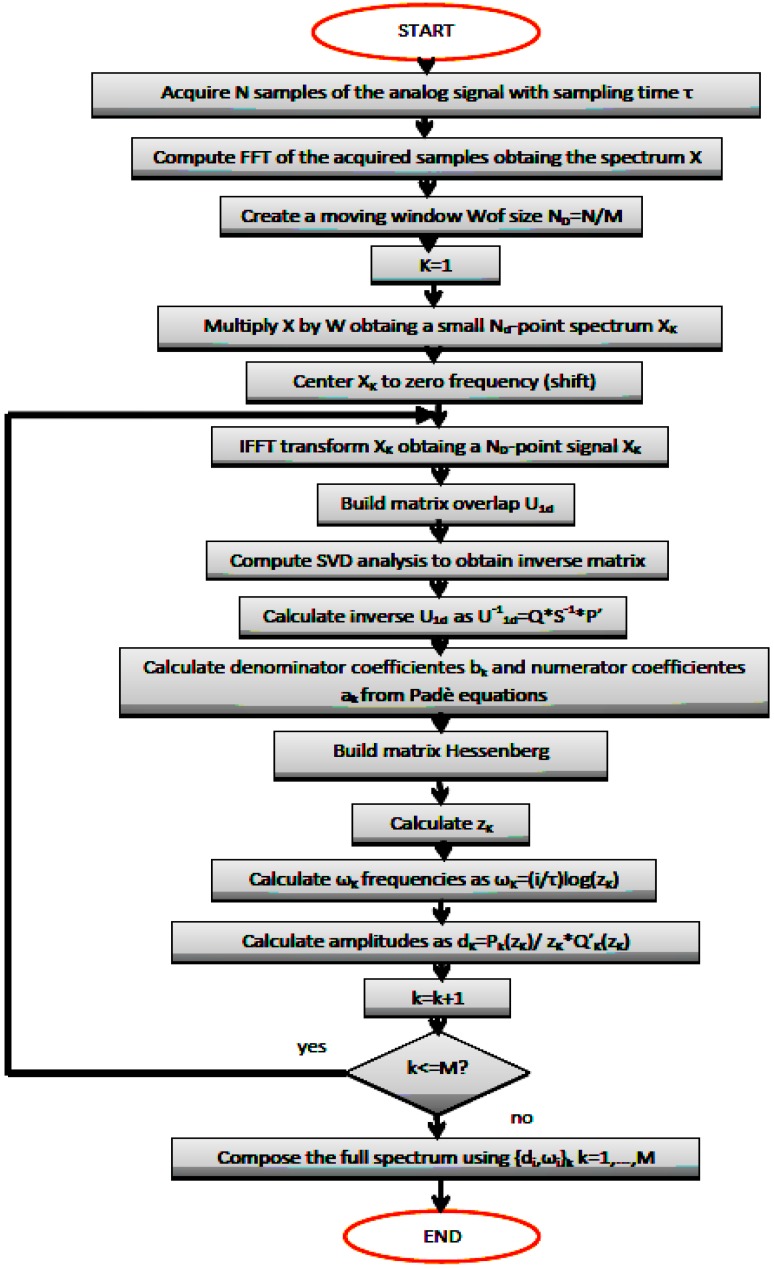
Concise DPA procedure for processing signal from pressure transducer.

Now we describe in a concise way the algorithms to be used for detecting the leaks within the pipeline. The acquisitions are simultaneous, and in automatic way the designed software allows to deliver outputs of both algorithms. A summary of DPA algorithm is illustrated in Figure 5. Formulae contained in this flowchart are explained in the previous section with more detailed. Afterwards, elements of DSD are also depicted.

**Figure 6:**
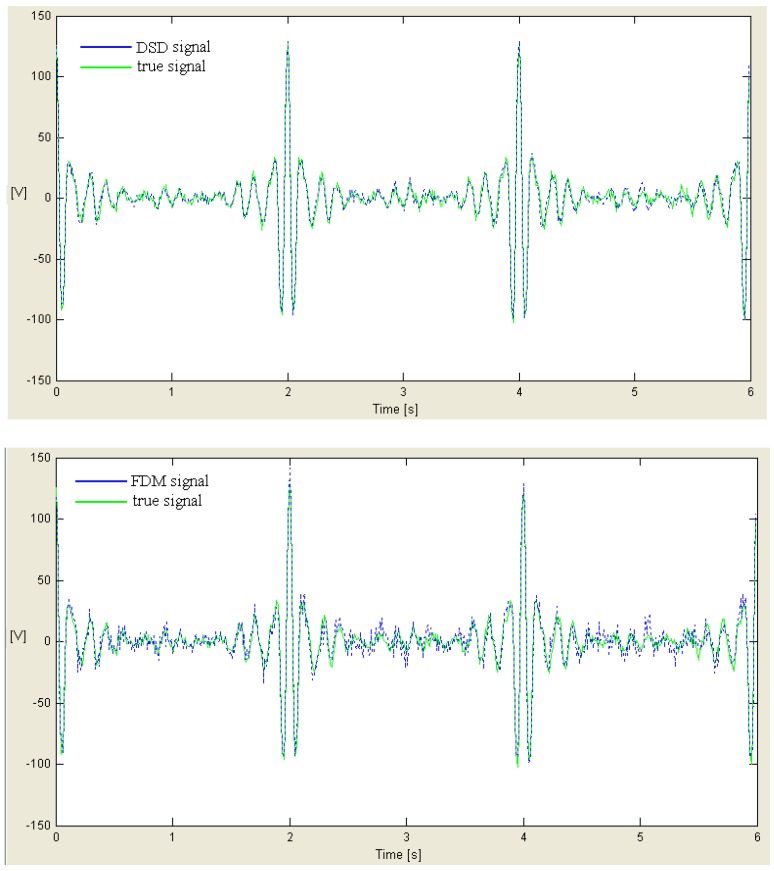
Comparison between processing effects of DSD and FDM of the same signal delivered by a pressure sensors since DSD partly includes FDM features.

Instructions performed by the procedure related to DSD algorithm are the followings:

1. A signal captured by pressure sensors, and processed by the hardware is supplied as *N* samples *c_n_* (digital signal) to be analyzed, and a value *τ* that must be considered as the temporal distance among samples (sampling interval);
2. *c_n_* is transformed using FFT, to obtain its low resolution spectrum:
  $$C_{k}=\sum_{n=0}^{N-1}c_{n}e^{j2\pi \frac{n}{N}k},\;\;k=0,\;\ldots ,\;N-1$$
3. The spectrum $\left\{C_{k}^{}\right\}$ is divided in *M* = *N*/*N_D_* parts or windows $\left[\omega_{k}{}_{\min},\;\omega_{k}{}_{\max}\right]$ each one containing $N_{D}=k_{\max}-k_{\min}+1$ samples of $\left\{C_{k}^{}\right\}$, by introducing a given overlapping among adjacent windows, eventually null;
4. Using samples of spectrum $\left\{C_{k}^{}\right\}$, it is possible to create *M* decimated spectra with limited bandwith ${\left\{C_{k}^{bld}\right\}}_{k=0,\ldots ,N_{D}-1}$, one for each window $\left[\omega_{k}{}_{\min},\;\omega_{k}{}_{\max}\right]$, taking only samples *C_k_* that are located within the corresponding window, and are translated for $\omega_{0}=\frac{\pi (k_{\max}+k_{\min})}{N\tau }=\frac{2\pi k_{0}}{N\tau }$, different for each window so that they can be centered at the origin ω=0;
5. Each spectrum $\left\{C_{k}^{bld}\right\}$ is processed using an inverse FFT (IFFT), by obtaining *M* decimated signals with limited bandwith ${\left\{c_{n}^{bld}\right\}}_{k=0,\ldots ,N_{D}-1}$; from point 6 up to 12, it is the same procedure as it is done in FDM except for the fact it must be repeated for each of the *M* signals $\left\{c_{n}^{bld}\right\}$. Figure 6 illustrates the effect of DSD and FDM on the same signal delivered by pressure transducer. It is necessary to be aware that the amplitudes have been magnified to allow a better understanding.
6. For each of the signals $\left\{c_{n}^{bld}\right\}$ it is necessary to construct square matrices $U_{0},U_{1}\in {\mathbb{C}}^{K,K}$ with *K* = [*N_D_*/2], and coefficients $U_{nm}=c_{n+m-1}^{bld}$ for $U_{1}$ and $U_{nm}=c_{n+m-2}^{bld}$ for $U_{0}$;
7. If $rank(U_{0})<K$ or the number of ill-posed steps of $U_{0}$ is so that $\rho (U_{0})\to +\infty$, then ${\bar{U}}_{0}=U_{0}^{+}$, where ${\bar{U}}_{0}$ has a diverse expression according to the adopted regularization technique, in particular:
  - for SVD it is $U_{0}^{+}=Q\Sigma^{+}P^{*}$ (Moore-Penrose pseudoinverse) [27];
  - for TSVD it is $U_{0}^{+}=Q_{t}\Sigma_{t}^{+}P_{t}^{*}$ [28];
  - for damped least squares, it is $U_{0}^{+}={\left(U_{0}^{*}U_{0}+q^{2}I\right)}^{-1}U_{0}^{*}$;
8. Needing to solve the problem $({\bar{U}}_{0}U_{1})B_{k}=u_{k}B_{k}$, searching for eigenvalues $u_{k}$ and eigenvectors $B_{k}$ of matrix ${\bar{U}}_{0}U_{1}$;
9. Eigenvectors are normalized $B_{k}$ so that $B_{k_{1}}^{T}U_{0}B_{k_{2}}=\delta_{k_{1}k_{2}}$;
10. An appropriate tolerance, γ>0, is established taking into account to keep the eigenvalues $u_{k}$ so that $1-\gamma <\left|u_{k}\right|<1+\gamma$, and the remaining are discarded;
11. Eigenvalues $\omega_{k}=-\frac{1}{\tau }∡(u_{k})$ are calculated, and amplitudes $d_{k}={\left(0|\omega_{k}\right)}^{2}={\left(C^{T}B_{k}\right)}^{2}$, with $C^{T}=\left[\begin{array}{ccc} c_{0}^{bld} & \ldots & c_{M-1}^{bld} \end{array}\right]$
12. For each window, borders of the spectrum are defined, by cancelling either right overlap or left one of the single window, that are, frequencies encompassed between the half of the portion of most external overlapped spectrum, by preserving those included in the most internal one;
13. Each spectrum is shifted by quantity $\omega_{0}$ computed at point 4, but in the opposite direction, in order to bring it in the original position;
14. Then, couples of parameters (*d_k_*, *ω_k_*) are created and computed for any sub-problem, and the spectrum $\sum_{k=1}^{K}d_{k}\delta \left(f+\frac{\omega_{k}}{2\pi }\right)$ is build.

**Figure 7:**
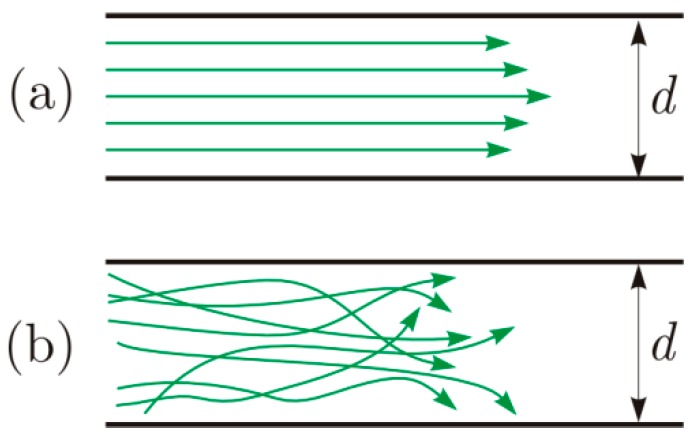
Detectable differences between laminar (**a**) and turbulent (**b**) within the pipeline.

Moreover, the pressure sensor does not only detect leakage phenomena but any variation of flow within the pipeline, and is able to discriminate laminar from turbulent flow as shown in Figure 7. Both regimes are possible according to conditions in which flow takes place, that are: pipe diameter, fluid velocity, roughness of inner wall of the pipe, and kinematic viscosity, that means:

(14) Re=V×Dν

in which *V* is the velocity in m/s, *D* the inner diameter of the pipe in meter, and the kinematic viscosity *ν* in m^2^/s. It is possible to establish the type of the flow in a pipe based on the value of the number of Reynolds [29]:

Re < 2000: laminar flow. In this condition, efforts due to viscosity are not influent.
Re > 4000: turbulent flow. The roughness of wall is important.
2000 > Re > 4000: transitional regime.

The Reynolds number allows one to determine the choice of piezoelectric sensor, since each device is related to an acquisition frequency that is the flow velocity. For the sensors of this paper, the frequency which data have been acquired is less than 3 Hz, and the burst pressure is less than 6 bar. We worked with pressure around 1 bar. The Reynolds number permitted us to choose a pressure sensor with a burst pressure that is not very high in order to be sensitive to pressure variations. However, since DSD, and FDM are used in nuclear resonance they are tailored for processing any frequency even high (for example values around GHz).

**Figure 8:**
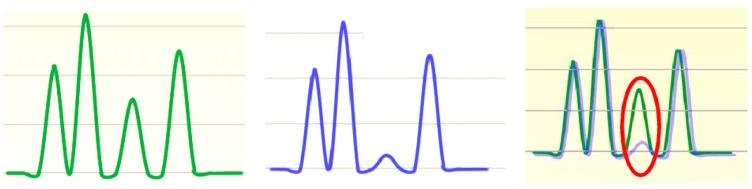
Mechanism of leak detection: theoretical spectrum (**left**), measured spectrum (**center**), and overlapping of both spectra to reveal leak (**right** in red).

The signal delivered by sensor contains the water pressure, and it is processed by means of a dedicated electronics containing a conditioning unit, and connected to an inverter that controls an electric pump. Then, the treated signal is received by a computer, through a DB9 female connector, capable of running DSD and FDM algorithms under Matlab environment by pinpointing the spectral differences between the modeled (or expected, or theoretical) spectrum and the measured one. The theoretical spectrum is here defined as the spectrum obtained without considering the time spent to open the water tap; it means zero delay, similar to Dirac delta function. Further details are included in [23]. Figure 8 illustrates the way of detecting a leak.

**Figure 9:**
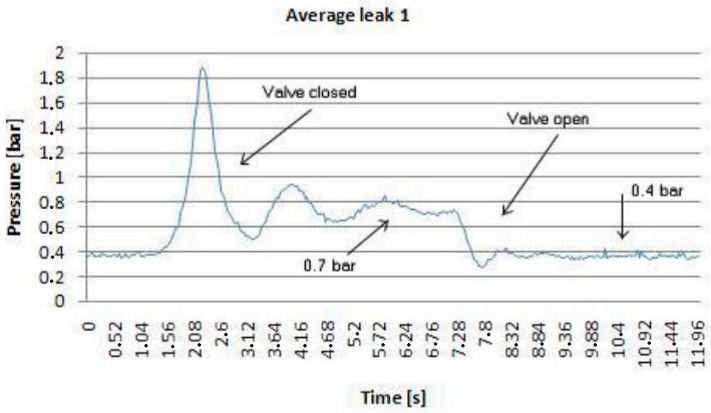
Correlation between different flow pressure trends by maneuvering water tap 1.

**Figure 10:**
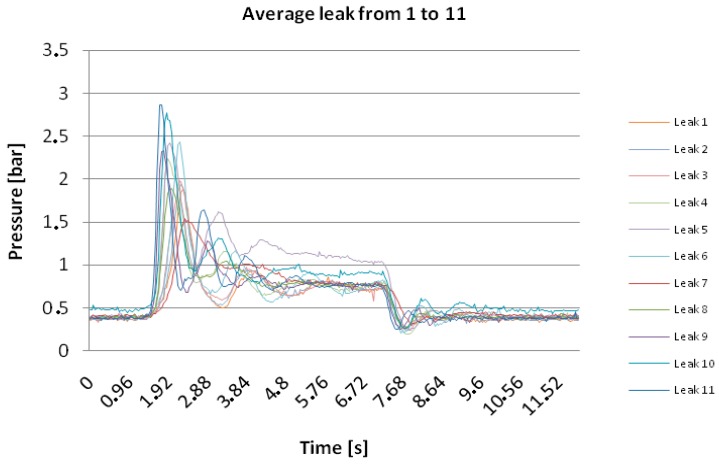
Correlation between different peaks and leak locations.

**Figure 11:**
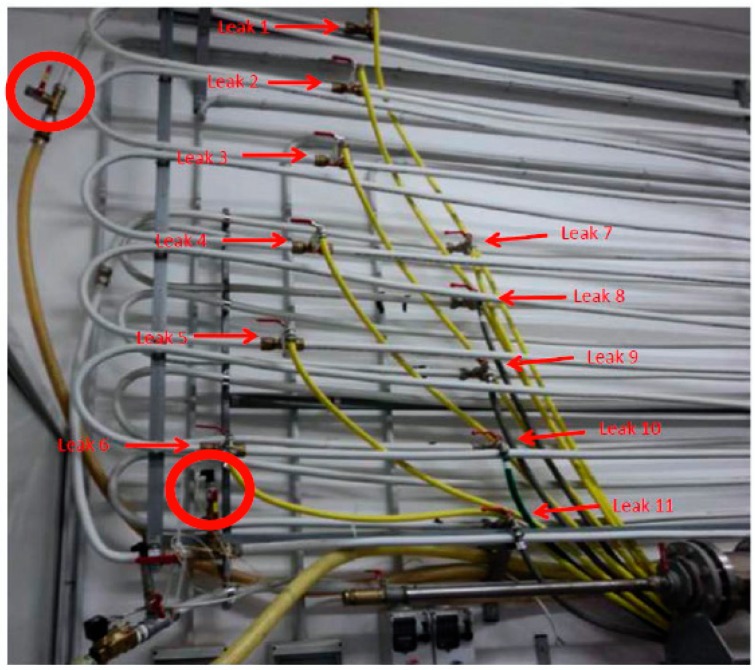
Leaks locations on the experimental pipeline with circled sensors.

Once to understand the flow regime is clear, as depicted in Figure 7, a series of tests are carried out regardless of the algorithm to be used after. The results for leak 1, for instance, opening and closing its water tap are demonstrated in Figure 9, while Figure 10 shows the different tests performed in order to establish a correlation between peaks at the valve (water tap) opening and closing as illustrated in Figure 11. The application of both algorithms are now simple.

The proposed algorithms process, in real-time, data received from the hydraulic plant by means of the dedicated hardware of Figure 3. All acquired data undergo a dedicated treatment using regression models and uncertainty determination as specified in [4]. An example of both algorithms spectra is shown in the next section. We have chosen separate frequency matching per algorithm in order to point out their peaks.

**Figure 12:**
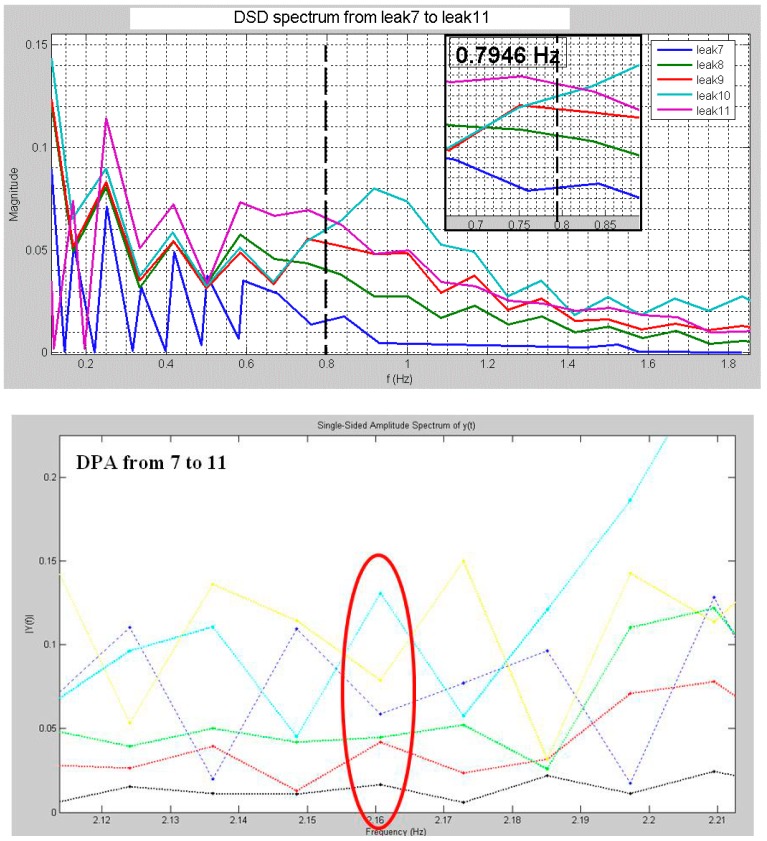
Detected peaks related to corresponding leaks for DSD and DPA respectively.

The results are illustrated in Table 1. Before commenting on them, it is useful to point out the following aspects: (i) the hydraulic plant, that is the pipeline, is divided in two parts (see also Figure 12), the first part including leaks from 1 up to 6, and the second one, located behind the first in upper position. So the second branch offers major piezometric state than the first branch; (ii) only one transducer covers the second branch since an additional sensor is located in the middle between both branches. Given the aforementioned considerations, we can notice DSD has less uncertainty, then more accurate, with the increasing of the work frequency that is the frequency allowing to detect the peak corresponding to the related leak. The uncertainty expressed in “±” meters is with respect to the position of the water tap that has been opened. In detail, when we open the first water tap (e.g.), from Table 1 we notice, for 1.2 Hz, DPA exhibits an uncertainty of ±6.42 m that is a range of 12.84 m around the leak; while DSD shows ±0.52 m that is a range of 1.04 m which the leak 1 is included in.

The performance of DPA is, instead, more different, that is in general good with the increasing of the work frequency, excepted for low frequencies (0.8 Hz) connected to high piezometric state. This is justified by the fact that in the second branch of the pipeline, there is an increasing of noise due to major friction of water at the piezometric state. DPA does not make a clear distinction between the friction frequency (noise) and peaks related to leaks within the pipe. The yellow-dashed areas of the Table 1 illustrate this behavior.

## 5. Conclusions and Final Outlook

In the effort to show the importance of improving the quality of information recovered from a pressure sensor mounted on a pipeline for SHM, the paper has presented a comparison between two innovative algorithms based on the decimated Padé approximant (DPA) and decimated signal diagonalization (DSD) techniques. They are used for monitoring the structure of an experimental zigzag pipeline. We have noticed that DPA is better when the noise contribution is not significant. DSD delivers results for short signals, that is, with less samples, and it is not expensive (computationally speaking) to extract spectral parameters due to the fact it is less subject to ill-posed problems; the time dedicated by DSD for computing FFT of the initial signal is around *N* log2*N*, with *N* intended as number of windows, is less than the total duration of the signal. The time dedicated to windowing is less influent, and can be considered negligible [30]. Most of the time for executing the algorithm is spent for resolving the problem of eigenvalues, and it varies considerably according to the regularization method used. It is beneficial to notice, from Table 1, DSD, with respected to common decimation that causes loss of information, preserves information contained in the signal, if we considered *N* windows. DSD offers the advantages of subdividing the problem in small ones. 

**Figure 13:**
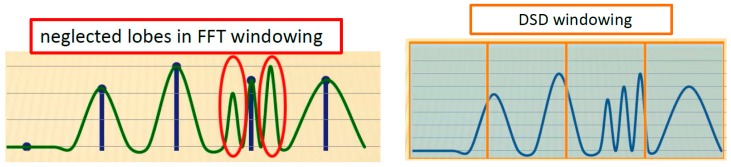
Effects of windowing based on FFT (**left**) and DSD (**right**).

For better understanding the effect of windowing, we recall the same spectrum processed by FFT and DSD. For FFT, when the signal has few samples, it is difficult to get a high resolution spectrum according to Figure 13 without a loss of information due to neglected lobes, but DSD exhibits better features. DPA and DSD are immune to noise with respect to FFT. DPA, DSD and FDM belong to the advanced transforms employed in nuclear magnetic resonance for processing complex signals. FFT is not mostly suitable for that. Of course, these advanced transforms are “*per se*” better than FFT since they face decaying processes in nuclear magnetic resonance. Quick changes and amount of information within the spectrum are a crucial issue for FFT. Many city waterworks operate with varying pressure and complicated architectures with the presence of piezometric towers, powerful suction pumps and eventual, and additional supply of water from wells. In these configurations, reducing uncertainty and increasing accuracy are a strategic goal. Figure 13 is a clear evidence of the importance of not losing precious information by working in time domain. 

The use of different frequencies to capture peaks related to leaks is an important way to understand the impact of sensor features in the overall process. The presented approach, in the effort to implement nondestructive tests with pressure sensors, can be extended to other infrastructures, deploying this double detection with two different algorithms in order to verify structural responses to diverse states. The control of waterworks by means of pressure sensors, and their signal processing, can be included in a wide approach of safeguarding environment, in particular hydrology [31,32] to be supervised by recent findings in the field of clouding and internet of things. 

## Figures and Tables

**Table 1:** Final results and comparison between both techniques in two work frequencies. The uncertainty is here intended as “±”, and data have been treated using linear regression.

	Work Frequency 1 [Hz]	DPA Uncertainty [m]	DSD Uncertainty [m]	Work Frequency 2 [Hz]	DPA Uncertainty [m]	DSD Uncertainty [m]
Leak 1	1.2	6.42	0.52	2.2	**5.61**	0.35
Leak 2	1.2	6.42	0.52	2.2	**5.61**	0.35
Leak 3	1.2	6.43	0.52	2.2	**5.62**	0.35
Leak 4	1.2	6.42	0.53	2.2	**5.72**	0.35
Leak 5	1.2	6.43	0.53	2.2	**5.72**	0.37
Leak 6	1.2	5.44	0.54	2.2	**5.72**	0.37
Leak 7	1.2	**5.02**	0.87	2.8	2.43	0.32
Leak 8	0.8	**5.10**	0.87	2.8	2.43	0.31
Leak 9	0.8	**5.10**	0.87	2.8	2.56	0.31
Leak 10	0.8	**5.02**	0.91	2.8	2.56	0.32
Leak 11	0.8	**5.02**	0.91	2.8	2.56	0.31

## References

[1] Lay-Ekuakille A., Palamara I., Caratelli D., Morabito F.C. (2013). Experimental Infrared Measurements for Hydrocarbon Pollutant Determination in Subterranean Waters. Rev. Sci. Instrum..

[2] Khulief Y., Khalifa A., Mansour R., Habib M. (2012). Acoustic Detection of Leaks in Water Pipelines using Measurements inside Pipe. J. Pipeline Syst. Eng. Pract..

[3] Lee P.J., Vítkovský J.P., Lambert M.F., Simpson A.R. (2005). Frequency Domain Analysis for Detecting Pipeline Leaks. J. Hydraul. Eng..

[4] Lay Ekuakille A., Vendramin G., Trotta G. (2010). Robust Spectral Leak Detection of Complex Pipelines using Filter Diagonalization Method. IEEE Sens. J..

[5] Brunner B. (2017). Piezoelectric Transducers for Structural Health Monitoring. www.isc.fraunhofer.de.

[6] De Almeida V.A.D., Baptista F.G., Mendes C.L., Budoya D.E. Experimental Analysis of Piezoelectric Transducers for Impedance-Based Structural Health Monitoring. Proceedings of the International Electronic Conference on Sensors and Applications.

[7] Duan W.H., Wang Q., Quek S.T. (2010). Applications of Piezoelectric Materials in Structural Health Monitoring and Repair: Selected Research Examples. Materials.

[8] Mohammadi V., Mohammadi S., Barghi F., Ebrahimi F. (2013). Piezoelectric Pressure Sensor Based on Enhanced Thin-Film PZT Diaphragm Containing Nanocrystalline Powders. Piezoelectric Materials and Devices—Practice and Applications.

[9] Ngo H.-D., Mukhopadhyay B., Ehrmann O., Lang K.-D. (2015). Advanced Liquid-Free, Piezoresistive, SOI-Based Pressure Sensors for Measurements in Harsh Environments. Sensors.

[10] Lay-Ekuakille A., Vergallo P., Griffo G. (2013). A Robust Algorithm based on DPA Technique for Processing Sensor Data in Leak detection in Waterworks. IET Sci. Meas. Technol..

[11] Lay-Ekuakille A., Vergallo P. (2014). Decimated Signal Diagonalization Method for Improved Spectral Leak Detection in Pipelines. IEEE Sens. J..

[12] Belkić D., Dando P.A., Taylor H.S., Main J. (1999). Decimated signal diagonalization for obtaining the complete eigenspectra of large matrices. Chem. Phys. Lett..

[13] Mandelshtam V.A., Taylor H.S. (1997). Harmonic Inversion of Time Signals and Its Application. J. Chem. Phys..

[14] Doost M.B. (2016). Resonant-state-expansion Born approximation with a correct eigen-mode normalisation. J. Opt..

[15] Szmytkowski R. (2003). Derivation of Schwinger variational principles. Phys. Lett. A.

[16] Gennarelli G., Al Khatib O., Soldovieri F. (2017). Inverse Source Data-Processing Strategies for Radio-Frequency Localization in Indoor Environments. Sensors.

[17] Pette O. (2004). A Fredholm Determinant Formula for Section Determinants of Bounded Operators. Integral Equ. Oper. Theory.

[18] Wu H.J. (2016). New Stone-Weierstrass Theorem. Adv. Pure Math..

[19] Jamieson M.J. (1987). Application of Wynn’s Epsilon Algorithm to Periodic Continued Fractions. Proc. Edinb. Math. Soc..

[20] Kalman D. A Singularly Valuable Decomposition: The SVD of a Matrix. The American University Washington. http://citeseerx.ist.psu.edu/viewdoc/download?doi=10.1.1.113.1193&rep=rep1&type=pdf.

[21] Yang S.-L. (2005). On the LU factorization of the Vandermonde matrix. Discret. Appl. Math..

[22] Tiwari H.M., Kumar Patel V., Mishra A. (2017). Application of Cauchy’s Residue Theorem to Solve Complex Integral using MATLAB. Int. J. Comp. Sci. Technol..

[23] Lay-Ekuakille A., Vendramin G., Trotta A. (2009). Spectral Analysis of Leak Detection in A Zigzag Pipeline: A Filter Diagonalization Method-Based Algorithm Application. Measurement.

[24] Adriana Savin A., Steigmann R., Bruma A., Šturm R. (2015). An Electromagnetic Sensor with a Metamaterial Lens for Nondestructive Evaluation of Composite Materials. Sensors.

[25] Tandeske D. (1990). Pressure Sensors: Selection and Applications.

[26] CVD Pressure Sensors. http://www.controlcomponents.com.au/products/documents/gems_pressure/gp07_gp09_Gems_CVDPsibar.pdf.

[27] Djordjević D.S., Dinčić N.C. (2010). Reverse order law for the Moore–Penrose inverse. J. Math. Anal. Appl..

[28] Bouhamidia A., Jbiloua K., Reichelb L., Sadoka H. (2011). An extrapolated TSVD method for linear discrete ill-posed problems with Kronecker structure. Linear Algebra Appl..

[29] Shankar Subramanian R. Pipe Flow Calculations. http://web2.clarkson.edu/projects/subramanian/ch330/notes/Pipe%20Flow%20Calculations.pdf.

[30] Karthik G.V.S., Yasmin Fathima S.K., Zia Ur Rahman M., Rafi Ahamed S.K., Lay-Ekuakille A. (2013). Efficient Signal Conditioning techniques for Brain activity in Remote Health Monitoring Network. IEEE Sens. J..

[31] Xiloyannis C., Dichio B., Celano G., Telesca V., Dalonzo F., Manfredi L. (2006). Drip irrigation of apricot orchards in areas with high evaporative demand and low rainfall: Accumulative effects on salinisation and some soil chemical characteristics. Acta Hortic..

[32] Scavone G., Sánchez J.M., Telesca V., Caselles V., Copertino V.A., Pastore V., Valor E. (2014). Pixel-oriented land use classification in energy balance modelling. Hydrol. Process..

